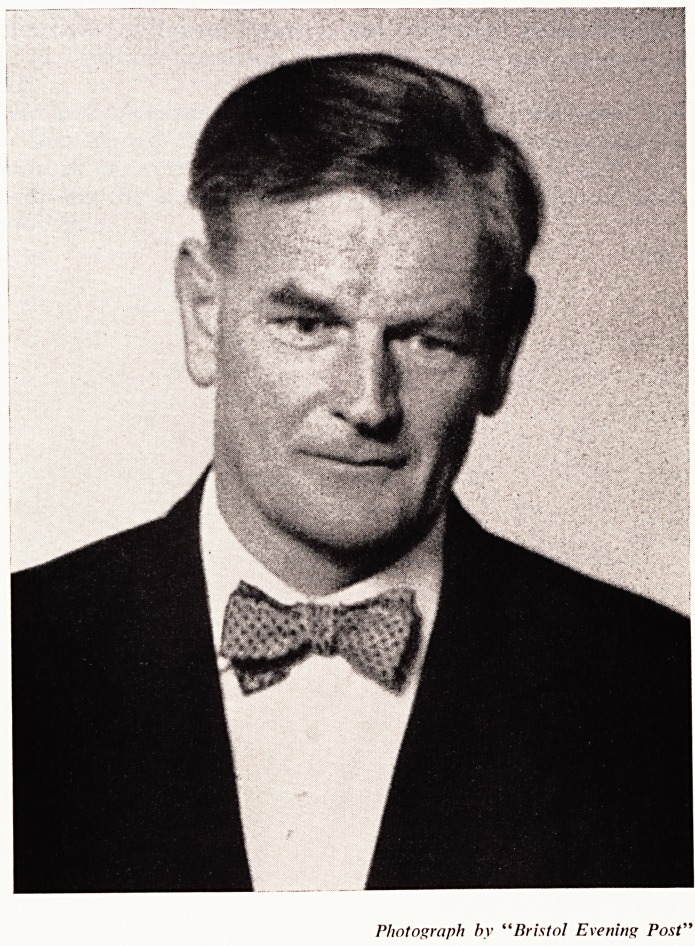# Kenneth Hampden Pridie

**Published:** 1963-07

**Authors:** 


### OBITUARY

### KENNETH HAMPDEN PRIDIE, M.B., B.S.(LOND.), F.R.C.S.

Mr. Kenneth Pridie, senior orthopaedic surgeon to the Bristol Royal Hospital and Winford Orthopaedic Hospital, and lecturer in orthopaedic surgery at the University of Bristol, died on 4th May 1963 at the age of 56 years while he was giving a paper at a meeting of the South West Orthopaedic Club at Exeter. Thus did we lose one of the most colourful and lovable characters on the contemporary orthopaedic stage.

K.P. grew up in Bristol, graduated in Medicine at Bristol University and the Bristol Royal Infirmary, and was indeed someone of whom Bristol was truly proud. His early interest in fractures and Orthopaedic Surgery was stimulated by working with Hubert Chitty and Ernest Hey Groves in Bristol, Girdlestone in Oxford, Watson-Jones in Liverpool, and Böhler in Vienna, and by 1934 he had started the Fracture Clinic at the Bristol Royal Infirmary and become the first surgeon in Bristol to devote himself wholly to orthopaedic surgery. He was only 27 years old when appointed Assistant Fracture Surgeon at the Bristol Royal Infirmary; his ability, his enthusiasm and boundless energy led to his early recognition in Bristol and in centres throughout the country as someone with an important contribution. In these early days he worked closely with Mr. Hey Groves, who had recently retired from the Bristol General Hospital, and Ken throughout his life retained an intense admiration for this great orthopaedic surgeon, to whose inspiration he always felt he owed much. K.P. took a leading part in the expansion of Winford Orthopaedic Hospital to serve the casualties of war and he extended his influence by setting up a number of outlying clinics at Bridgwater, Burnham-on-Sea and Tetbury.

His originality of thought, vivid personality, and charm led to his becoming widely known throughout the country and many parts of the world; his contributions at orthopaedic meetings were always striking and resulted in Bristol receiving many orthopaedic visitors. His work on fractures and osteoarthritis of the hip and knee was best known but his originality in devising operations and instruments has left a permanent imprint on orthopaedic surgery. In spite of his formidable physique he was a neat, delicate and quick operator, often quite brilliant and showing considerable courage, which is inseparable from innovation in surgery. K.P. was a forthright and colourful speaker and with his pleasant wit and aptitude for quotation he never failed to captivate his audience, and a smattering of overstatement would only whet the appetite. He contributed a good deal to the literature but could have written more to our great benefit.

In the world of sport Ken Pridie had a distinguished career. He was University heavy-weight boxing champion in 1925–26, he was a prominent forward in the Bristol Rugby Club for many years, but it was in shot putting and discus that he was pre-eminent. In the latter he broke the British native record in 1931 and he represented England in both the shot putting and discus in the Empire Games in 1930 and 1934 and was selected for the Olympic Games in 1932 although he was unfortunately unable to attend.

With all his attributes, professional and athletic, those who knew him will remember him best as a man, full of ideas and enthusiasm, an individualist; one felt better for being with him and absorbing some of his infectious energy. He had a great humility and his sense of fun endeared him to the children of all his friends and contributed much to the happiness of his home and large family, of which he was so proud. He was a man of forceful personality and definite ideas, but capable of being persuaded

*Photograph by "Bristol Evening Post"*

if your case was a convincing one. He was free from malice to an unusual degree and he took success or failure with the courage of a great sportsman. His tastes were simple; outdoor activities whether at "The Chalet" or on the Scilly Isles with his family and friends all round him provided the relaxation he loved.

The Bristol Medico-Chirurgical Society has lost a member who attended its meetings with great regularity—so wide were his interests—and who enlivened the meetings with his devastatingly simple comments.

In 1934 he married Dr. Joanna Egerton and with their seven children they have shown to many in Bristol a home and family life that it was always a pleasure and privilege to enter.

A very wide circle of friends in many walks of life bemoan the loss of a great character—in the very best sense of the word—and a man different from and with a greater capacity than most of us, in that there was a touch of genius in K.P. As a colleague one could not help but derive outstanding benefit—ideas, instruction and inspiration—from Kenneth Pridie.

A. L. E-B.

## Figures and Tables

**Figure f1:**